# Lipid Content and Composition during the Oocyte Development of Two Gorgonian Coral Species in Relation to Low Temperature Preservation

**DOI:** 10.1371/journal.pone.0038689

**Published:** 2012-07-27

**Authors:** Chiahsin Lin, Li-Hsueh Wang, Tung-Yung Fan, Fu-Wen Kuo

**Affiliations:** 1 National Museum of Marine Biology & Aquarium, Checheng, Pingtung, Taiwan; 2 Graduate Institute of Marine Biotechnology, National Dong Hwa University, Checheng, Pingtung, Taiwan; 3 Graduate Institute of Marine Biodiversity and Evolution, National Dong Hwa University, Checheng, Pingtung, Taiwan; Baylor College of Medicine, United States of America

## Abstract

Our previous studies have suggested that chilling sensitivity of coral oocytes may relate to their relatively high lipid intracellular content and lipid composition. The distribution of lipids during the oocyte development was determined here for the first time in two gorgonian species (*Junceella juncea* and *Junceella fragilis*). The main lipid classes in the two gorgonian oocytes were total lipid, wax ester, triacylglycerol, total fatty acid, phosphatidylethanolamine and phosphatidylcholine. The results indicated that early stage oocytes of *J. juncea* and *J. fragilis* were found to have increased lipid content than late stage oocytes. The content of wax ester was significantly higher in the early stage oocytes of two gorgonian corals (51.0±2.5 and 41.7±2.9 µg/mm^3^/oocyte) than those of late stage oocytes (24.0±1.4 and 30.4±1.2 µg/mm^3^/oocyte, respectively). A substantial amount of phosphatidylethanolamine and total fatty acid was detected at each stage of oocyte development in two gorgonian ranges from 107 to 42 µg/mm^3^/oocyte and 106 to 48 µg/mm^3^/oocyte, whilst low levels of phosphatidylcholine were found in two gorgonian oocytes. The levels of total lipid in the late stage oocytes of *J. juncea* were significantly higher than those of *J. fragilis*. The observed differences may partially be related to different habitat preferences as higher lipid levels in *J. juncea,* a deeper-water coral species exposed to lower temperature seawater, might relate to adjustments of cell membranes in order to increase membrane fluidity.

## Introduction

Gorgonian corals are suffering continuing decline in population size and reproductive ability due to environmental stresses such as pollution, habitat destruction and global climate change [1]. Cryopreservation technologies are urgently needed to establish conservation measures to preserve coral populations. Cryopreservation of coral sperm has been successful [2]. However, chilling sensitivity of coral larvae has been reported to be very high [3]. When the temperature was below 10°C, coral larvae showed membrane damage with short exposure and there was no larvae survival at −11°C [3]. Studies on the cryobiology of coral oocytes have been carried out in our laboratory [4], [5], [6], [7]. We have reported that hard coral (*Echinopora* spp.) and gorgonian coral (*J. juncea* and *J. fragilis*) oocytes showed significant levels of chilling tolerance at 5°C and 0°C, however, these oocytes were very sensitive to chilling at −5°C resulting in a significant decline in ATP concentration after 4 h chilling [6], [7].

In some mammalian species, the high chilling sensitivity of porcine and bovine oocytes is related to their high intracellular lipid level [8], [9], [10]. Research on porcine and bovine embryos has demonstrated that less lipid accumulation in embryos appears to be highly associated with an increased embryo survival during cryopreservation procedures [11], [12]. The high lipid content has also been linked to chilling sensitivity in zebrafish embryos and ovarian follicles [13], [14]. In coral oocytes (*Stylophora pistillata*), the lipid accumulation increases during maturation of the oocytes and lipid content remains high until spawning [15]. Our previous studies have suggested that sensitivity of coral oocytes to lower temperatures may relate to their relatively high lipid intracellular content and/or lipid composition as these oocytes were collected during the spawning season [6], [7]. To address the relationship between lipid and cryosensitivity in corals, the present study set out to investigate the composition of the total lipid content, neutral lipid content (wax ester and triacylglycerol), total fatty acid and polar lipids (phosphatidylethanolamine and phosphatidylcholine) in two different oocyte developmental stages of gorgonian corals.

## Results

### Lipid distribution in the oocytes of two gorgonian species

Early stage oocytes of *J. juncea* had an average volume of 0.0054 mm^3^ slightly smaller than that of *J. fragilis* oocytes (0.0066 mm^3^). However, the oocyte volume increased during oogenesis and late oocytes had an average volume of 0.0137 mm^3^ and 0.0160 mm^3^ respectively. The percentages of individual lipid classes in early and late oocytes of two gorgonian corals are shown in Table 1. The main lipid classes in coral oocytes were wax ester, triacylglycerol, total fatty acid, phosphatidylethanolamine and phosphatidylcholine. The same lipid classes were detected in early and late stage oocytes of two gorgonian corals. The main lipid components in the early and late stage oocytes of *J. juncea* were identified as total fatty acid (36.4% and 58.0%, respectively) followed by phosphatidylethanolamine (36.9% and 23.3%), wax ester (17.7% and 13.1%), phosphatidylcholine (8.9% and 5.5%) and triacylglycerol (<1%). However, in early and late oocytes of *J. fragilis* a higher level of phosphatidylethanolamine was obtained with 54.4% and 43.8%, respectively in comparison to the other lipid classes with total fatty acid (24.4% and 37.7%), phosphatidylethanolamine (21% and 14%), phosphatidylcholine (<1% and 4.6%) and triacylglycerol (<1%).

**Table 1:** Wax ester (WE), triacylglycerol (TAGs), total fatty acid (TFA), phosphatidyethanolamine (PE) and phosphatidylcholine (PC) content of oocyte of two gorgorian corals.

	*J. juncea*		*J. fragilis*	
	Early stage	Late stage	Early stage	Late stage
Oocyte volume (mm^3^)	0.0054±0.0004	0.0138±0.0012	0.0066±0.0004	0.0160±0.0008
WE (%)	17.7	13.1	21.1	13.8
TAGs (%)	<1.0	<1.0	<1.0	<1.0
TFA (%)	36.4	58.1	24.4	37.7
PE (%)	36.9	23.3	54.4	43.8
PC (%)	8.9	5.5	<1.0	4.6

Data are % composition of total lipid.

### Effect of different coral stages on lipid composition in two gorgonian species

**Figure 1:**
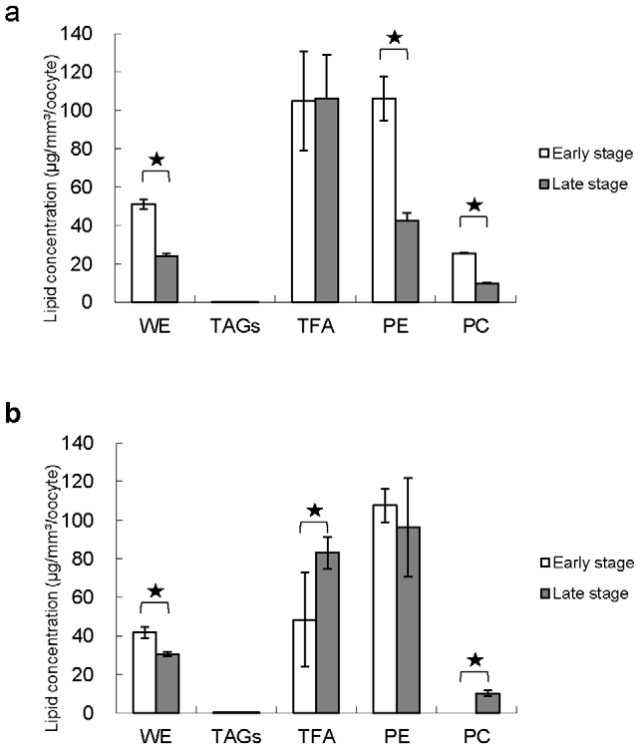
The distribution of wax ester (WE), triacylglycerol (TAGs), total fatty acid (TFA), phosphatidyethanolamine (PE) and phosphatidylcholine (PC) extracted from early and late stages oocytes of *J. juncea* (a) and *J. fragilis* (b) oocytes. Error bars indicate standard errors of the means. Asterisks represent significant difference between of the same lipid category between early and late stage oocytes (*p<0.05*).

Figure 1 displayed the lipid composition of different stage oocytes in two gorgonian species. The content of wax ester was significantly (*p<0.05*) higher in the early stage oocytes of two gorgonian corals (51.0±2.5 and 41.7±2.9 µg/mm^3^/oocyte) than that of late stage oocytes (24.0±1.4 and 30.4±1.2 µg/mm^3^/oocyte, respectively (Fig. 1a, 1b). In contrast, higher level (*p<0.05*) of total fatty acid was found in the late stage oocytes of *J. fragilis* (83.0±8.2 µg/mm^3^/oocyte) than in the early stage oocytes (48.3±24.5 µg/mm^3^/oocyte, Fig. 1b), whilst there were no significant (*p>0.05*) differences in the contents of total fatty acid between the early and late stage oocytes of *J. juncea* (Fig. 1a). A substantial amount of phosphatidylethanolamine was detected at each stage of oocyte development range from 42 to107 µg/mm^3^/oocyte, whilst relatively low levels of phosphatidylcholine were found in all oocytes (Fig. 1a, 1b). The content of phosphatidylethanolamine was significantly (*p<0.05*) higher in early stage oocytes of *J. juncea* (106.3±11.6 µg/mm^3^/oocyte) than that of late stage oocytes (42.5±4.1 µg/mm^3^/oocyte, Fig. 1a). There were no significant (*p>0.05*) differences in the abundance of phosphatidylethanolamine at each developmental stage in oocytes of *J. fragilis* with 107.6±8.7 and 96.3±25.6 µg/mm^3^/oocyte, respectively (Fig. 1b).

### Effect of two gorgonian species on lipid composition

**Figure 2:**
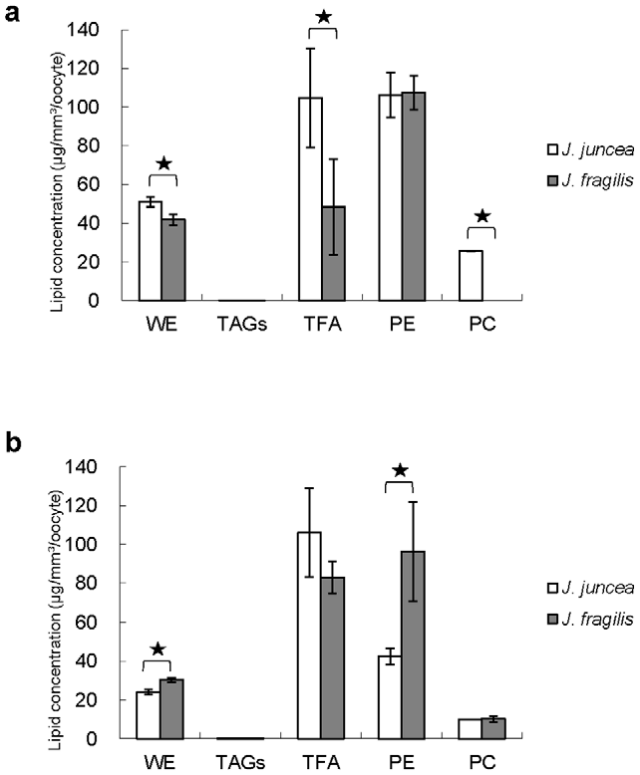
The composition of lipid content in early (a) and late (b) stage oocytes of *J. juncea* and *J. fragilis* oocytes. Error bars indicate standard errors of the means. Asterisks represent significant difference of the same lipid category between *J. juncea* and *J. fragilis* oocytes (*p<0.05*).

The effects of two gorgonian species on lipid composition are shown in Figure 2. The largest component was total fatty acid, phosphatidylethanolamine and wax ester in the oocytes of two gorgonians (Fig 2a, 2b). The concentration of wax ester and total fatty acid was significantly (*p<0.05*) higher in early stage oocytes of *J. juncea* (50.9±2.5 and 104.8±25.7 µg/mm^3^/oocyte) than that of oocytes of *J. fragilis* with 41.7±2.9 and 48.3±24.5 µg/mm^3^/oocyte, respectively (Fig. 2a). The greater abundance of phosphatidylethanolamine was not statistically different between early stage oocytes of two gorgonian species (Fig. 2a). In contrast to early stage, the level of phosphatidylethanolamine was significantly higher in late stage oocytes of *J. fragilis* than *J. juncea* oocytes (Fig. 2b). The concentration of wax ester was significantly lower in late stage oocytes of *J. juncea* (24.0±1.4 µg/mm^3^/oocyte) than that of oocytes of *J. fragilis* (30.4±1.2 µg/mm^3^/oocyte, *p>0.05*), whilst there were no statistical (*p>0.05*) differences in the larger amounts of TFA in two gorgonian species with 106.0±22.9 and 83.0±8.1 µg/mm^3^/oocyte, respectively (Fig. 2b).

### Total lipid concentration in two gorgonian species

**Figure 3:**
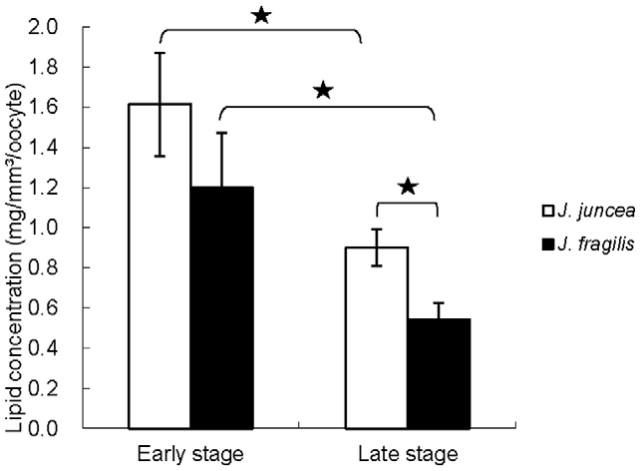
The distribution of total lipid in early and late stage oocytes of *J. juncea* and *J. fragilis.* Error bars indicate standard errors of the means. Asterisks represent significant difference between groups (*p<0.05*).

Figure 3 showed total lipid concentration in the oocytes of the two gorgonian species. Total lipid concentrations in late stage oocytes of two gorgonian species were lower than those of early stage oocytes (Fig. 3). The level of total lipid in the late stage oocytes of *J. fragilis* were significantly lower (0.5±0.1 µg/mm^3^/oocyte, *p<0.05*) than those of *J. juncea* (0.9±0.1 µg/mm^3^/oocyte), whilst there were no significant (*p>0.05*) differences in the contents of total lipid in the early stage oocytes of *J. juncea* (1.2±0.4 and 1.6±0.6 µg/mm^3^/oocyte, Fig. 3).

## Discussion

Numerous studies have shown an increasing interest in lipid biology and biochemistry of corals [18], [19], [20], [21]. Aside from structural functions in cell membranes, early studies on coral species have reported that lipids serve as an energy store in coral species for processes involved in tissue growth [18], [22], skeletal growth [23], and reproduction [24]. It has been reported that total lipid at concentrations of 10–40% of dry biomass was observed in a number of corals from tropical seas [18], [20], [21]. It has also been shown that some coral species maintain enough lipids to sustain their metabolic energy requirements for up to 114 days, when symbiotic algae does not supply the coral with fixed carbon by photosynthetic processes under conditions of insufficient sunlight [25], [26]. Direct observation by light microscopy has revealed that coral eggs contain numerous lipid droplets occupying around 80% of the volume of the egg [27]. In the present study, total lipid content decreased with oocyte developmental stages in two gorgonian species indicating the potential utilization of these nutrients as an energy source by oocytes such as new cell constitution and organogenesis as well as for energy production. Our present study has also found that late stage *J. juncea* oocytes contained higher levels of total lipid when compared to *J. fragilis* and the observed differences may partially be related to different habitat preferences. The higher lipid levels found in *J. juncea* suggest that in deeper-water coral the large amount of lipids might be related to adjustments of cell membranes in order to increase membrane fluidity.

**Table 2:** Gradient elution program for HPLC-ELSD separation.

Time (min)	solvents			Flow rate (ml/min)
	A(%)	B(%)	C(%)	
0	100	0	0	1.0
4	100	0	0	1.0
5	85	15	0	1.0
10	80	20	0	1.0
12	75	25	0	1.0
15	50	50	0	1.0
18	30	50	20	1.0
20	30	40	30	1.0
25	25	30	45	1.0
30	30	70	0	1.0
40	100	0	0	1.0

The main storage lipids were considered to be wax esters and triacylglycerols as the concentration of storage lipids accounted for range of 40–73% of total lipids in corals [20], [21], [28], [29], [30]. Moreover, concentrations of storage lipids have been described to fluctuate in response to coral metabolic requirements [29], [31], reproductive rate [19], egg production [32] and zooxanthellae productivity [19], [33], [34]. It has been reported that storage lipid accounted for between 46% and 73% of the total lipid in coral species such as *Pocillopora capitata* and *P. verrucosa*, the major component of the storage lipid being triglyceride [20], [33]. Triacylglycerols have been described as the main lipid classes found in marine invertebrates. They are involved in the oocyte maturation and the initial larval survival in marine invertebrates [35], [36], [37], [38], [39]. However, the lipid composition in gorgonian corals is different from those of other coral species. In the present study, triacylglycerols were found less than 1% of total lipid in both stages of two gorgonian oocytes. Although wax esters were not found in lipid content of mammalian cells [40], they were present in the gorgonian oocytes with a substantial concentration up to 13% in the early and late stages. Oocytes with high concentration of lipid have been reported in the some coral species such as the *Alcyonium glomeratum* [41] and *Stylophora pistillata* [15] where lipid content increase during oocyte maturation and remains high until spawning [15]. Lipid levels in coral eggs have showed a high proportion of wax esters to work as a buoyancy substance and as energy reserves [32], [42]. A similar result was also seen in two gorgonian coral species in the present study as wax esters are considered to act as an energy source for gorgonian eggs.

Fatty acids provide a valuable energy source to coral species [30], with saturated and monounsaturated fatty acids stored as wax esters [29], [43]; as membrane components in the form of phospholipids, and as polyunsaturated fatty acids (PUFAs) [44], [45] influencing reproduction and membrane fluidity [46]. In the present study total fatty acid was identified as the main lipid components of the gorgonian oocytes. These two gorgonian coral oocytes are characterized by the presence of higher levels of total fatty acid in *J. juncea* and a lower composition in *J. fragilis*. In fact, with increasing depth, there appears to be an increase in pressure as well as a decrease in temperature. Therefore, the higher level of total fatty acid in *J. juncea* oocytes may help to increase membrane fluidity at lower depths. The essential fatty acids start with the short chain polyunsaturated fatty acids that coral hosts are incapable of constructing essential fatty acids due to an inability to synthesize from endogenous production; additional amounts must be procured through their diet to gain the required precursors [47], [48]. *Montipora digitata* oocytes acquire symbiotic zooxanthellae by maternal inheritance and various hard coral *Montipora* species may contain 102–103 zooxanthellae in an egg at the time of spawning [49]. Symbiotic zooxanthellae undergo division during embryogenesis of coral host and are capable of translocating carbon compounds to the host during early development [32]. Early studies also showed that corals containing high levels of unsaturated fatty acids relied more on plankton capture, whilst corals containing greater amount of saturated fatty acids relied more on the translocation of photosynthetic products from the zooxanthellae [50]. In our present study, *J. fragilis* oocytes inherits zooxanthellae and contained less total fatty acid than *J. juncea* oocytes which carry no symbiotic zooxanthellae. It is possible that fatty acid profiles of lipids were influenced by the presence or absence of algal symbionts in gorgonian oocyte and may lead to a significant difference in fatty acid compositions. Studies are currently under way in our laboratory in this area.

It is well-established that biological cellular membranes are consisted mostly of amphipathic phospholipids, such as phosphatidylethanolamine and phosphatidylcholine which are predominantly located in marine invertebrate [51]. Within the phospholipid class, phosphatidylcholine seems to be the major component, followed by phosphatidylethanolamine in fish eggs [52], [53]. However, some marine invertebrate such as sponges, soft corals, and molluscs may produce more phosphatidylethanolamine than phosphatidylcholine. The proportion of phosphatidylethanolamine is over 60% and even more than 80% of the total phospholipids in some of these animals [51]. The result obtained in this study is in agreement with a previous study which also showed phosphatidylethanolamine was the main phospholipids and contains more phosphatidylethanolamine than phosphatidylcholine for soft corals [51].

The results of this study provided a detailed quantitative account of the lipid composition of *J. juncea* and *J. fragilis* oocytes. In our study, gorgonian corals living in the depth range from 3 to 35 m had significant difference in lipid content and compositions. The results indicated that early stage gorgonian oocytes contained more lipid content than late stage oocytes. Higher percentages of lipid content were observed in *J. juncea*. Our previous studies have found that oocytes of *J. juncea* are less chilling sensitive than *J. fragilis* oocytes, due to their deeper natural habitat and their high intracellular lipids level of these oocytes is probably responsible for their high cryosensitivity [7]. The higher lipid contents in oocytes of *J. juncea* suggest that the deeper-water species and lower temperature seawater might have promoted accumulation of lipids related to biochemical adjustments of cell membranes to increase membrane fluidity. The result of the present study clearly demonstrated that the high sensitivity of two gorgonian oocytes to low temperature is related to their high lipid content.

## Materials and Methods

### Collection of *J. juncea* and *J. fragilis*

*J. juncea* and *J. fragilis* were collected during the reproductive season from July to September 2009. The corals were collected by scuba diving in a depth range of 3 to 30 m in Kenting National Park, Nanwan, Taiwan (21°56′N, 120°44′E). The *J. fragilis* colonies were found on the seaward slopes at a depth range of 3 to 10 m, whilst *J. juncea* colonies settle below 20 m in depth. Both corals were cut into branches (about 60 cm in length) using a pair of surgical scissors. After collection, the coral branches were kept in a 200 L container with native seawater and then transported immediately to the Coral Husbandry Center, National Museum of Marine Biology & Aquarium with a seawater flow system at 25°C. The coral collection was approved by Kenting National Park Management Office.

### Oocyte isolation

Coral coenchyme tissues were removed from coral branches using a scalpel and were immediately transferred into 6-well tissue culture dishes with 2 ml filtered (0.4 µm) natural seawater (35 part per thousand). Oocytes were separated mechanically from the coenchyme tissue using forceps and pipette sucking. Oocyte isolation was carried out under a dissecting microscope (Olympus, SZ51, US). Oocytes were washed three times with filtered natural seawater and then kept in the filtered natural seawater for further processing. The developmental stages of the gorgonian oocytes were classified based on their size (Tsai et al., 2010). The diameters of the oocytes were measured with an ocular micrometer under the microscope. The sizes of early stage oocytes were in the *range* of 100 to 200 μm and late stage oocyte ranged from 200 to 300 μm. Oocytes of these two stages were used in the present studies.

### Lipid analysis

Total lipids were homogenized and extracted from 50 oocytes of *J. juncea* and *J. fragilis* in chloroform: methanol (2∶1) following the method described by Bligh and Dyer [16]. Lipid classes were separated by high performance liquid chromatography with evaporative light scattering detector (HPLC-ELSD). The extracted lipids were normalized for oocyte volume and number of oocytes and analyzed by HPLC-ELSD [17]. A Hitachi Model L7100 HPLC pump was connected with a Sedex 80 evaporative light-scattering detector (Sedex, France). The system was also equipped with an autosampler (Hitachi, L7200, Japan). An YMC-PVA-SIL column (100 X3 mm i.d.; 5 mm particles; Hichrom Ltd, UK) was used for separations. The solvent was evaporated with nitrogen gas. The ELSD drift tube and nebulisation temperatures were maintained at 55°C and the flow rate of the nebulizer gas was set at 2.5 kg/cm^2^. The gradient elution program was shown in Table 2.

### Statistical analysis

Each treatment in the experiment contained three replicates and experiments were repeated at least three times. The statistical analysis was performed using the SPSS software (Version 17.0; SPSS Inc., Chicago, IL, USA). The data were checked for normal distribution with the one-sample Kolmogorov-Smirnov test and the variances with the Levene's test for homogeneity. Differences between the three different groups were tested using a One-way ANOVA of variance followed by Tukey's multiple comparison tests. In all statistical tests used, P values of less than 0.05 were considered to be significant. Results are presented as means ± SEM.

## References

[1] Blair AC (2003) Phenotypic variation and plasticity in *Leptogorgia virgulata,* Master's thesis, College of Charleston, Charleston, South Carolina.

[2] Hagedorn M, Carter VL, Steyn RA, Krupp D, Leong JC, et al (2006a) Preliminary studies of sperm cryopreservation in the mushroom coral, *Fungia scutaria* . Cryobiology 52: 454–458. 16626677 10.1016/j.cryobiol.2006.03.001

[3] Hagedorn M, Pan R, Cox EF, Hollingsworth L, Krupp D, et al (2006b) Coral larvae conservation: Physiology and reproduction. Cryobiology 52: 33–47. 16337183 10.1016/j.cryobiol.2005.09.008

[4] Tsai S, Spikings E, Haung IC, Lin C (2010a) Study on the mitochondrial activity and membrane potential after exposing later stage oocytes of two gorgonian corals (*J. juncea* and *J. fragilis*) to cryoprotectants. Cryo Lett 32: 1–12. 21468448

[5] Tsai S, Spikings E, Kuo FW, Lin C (2010b) Use of an adenosine triphosphate assay, and simultaneous staining with fluorescein diacetate and propidium iodide, to evaluate the effects of cryoprotectants on hard coral (*Echinopora* spp.) oocytes. Theriogenology 73: 605–611. 20005561 10.1016/j.theriogenology.2009.10.016

[6] Lin C, Tsai S (2011) The effect of chilling and cryoprotectants on hard coral (*Echinopora* spp.) oocytes during short-term low temperature preservation. Theriogenology. In press. 10.1016/j.theriogenology.2011.09.021 22153264

[7] Lin C, Zhang T, Kuo FW, Tsai S (2011) Studies on oocytes chilling sensitivity in the context of ATP response of two gorgonian coral species (*J. juncea* and *J. fragilis*). CryoLetters 32: 141–147. 21766143

[8] Nagashima H, Kashiwazaki N, Ashman RJ, Grupen CG, Seamark RF, et al (1994) Removal of cytoplasmic lipid enhances the tolerance of porcine embryos to chilling. Biol Reprod 51: 618–622. 7819441 10.1095/biolreprod51.4.618

[9] Leibo SP, Pollard JW, Martino A (1995) Chilling and freezing sensitivity of “reassembled”in vitro-derived bovine embryos. Theriogenology 43: 265–265.

[10] Martino A, Pollard JW, Leibo SP (1996) Effect of chilling bovine oocytes on their developmental competence. Mol Reprod Dev 45: 503–512. 8956289 10.1002/(SICI)1098-2795(199612)45:4<503::AID-MRD13>3.0.CO;2-X

[11] Dobrinsky JR (2001) Cryopreservation of swine embryos: a chilly past with a vitrifying future. Theriogenology 56: 1333–1344. 11758887 10.1016/s0093-691x(01)00634-3

[12] Seidel Jr GE (2006) Modifying oocytes and embryos to improve their cryopreservation. Theriogenology 65: 228–235. 16263160 10.1016/j.theriogenology.2005.09.025

[13] Liu XH, Zhang T, Rawson DM (2003) Effects of methanol and developmental arrest on chilling injury in zebrafish (*Danio rerio*) embryos. Theriogenology 59: 1545–1556. 12559459 10.1016/s0093-691x(02)01199-8

[14] Tsai S, Rawson DM, Zhang T (2009) Studies on chilling sensitivity of early stage zebrafish (*Danio rerio*) ovarian follicles. Cryobiology 58: 279–286. 19233154 10.1016/j.cryobiol.2009.02.002

[15] Oku H, Yamashiro H, Onaga K, Sakai K, Iwasaki H (2003a) Seasonal changes in the content and composition of lipids in the coral *Goniastrea aspera* . Coral Reefs 22: 83–85.

[16] Christie W, Gill S, Nordback J, Itabashi Y, Sanda S, et al (1998) New procedures for rapid screening of leaf lipid components from Arabidopsis. Phytochemical Analysis 9: 53–57.

[17] Bligh EG, Dyer WJ (1959) A rapid method of total lipid extraction and purification. Can J Biochem Physiol 37: 911–917. 13671378 10.1139/o59-099

[18] Battey JF, Patton JS (1984) A reevaluation of the role of glycerol in carbon translocation in zooxanthellae-coelenterate symbiosis. Mar Biol 79: 27–38.

[19] Stimson JS (1987) Location, Quanity and rate of change in quantity of lipids in tissue of Hawaiian Hermatypic corals. Bull Mar Sci 41: 889–904.

[20] Harland AD, Navarro JC, Davies PS, Fixter LM (1993) Lipids of some Caribbean and red sea corals: total lipid, wax esters, triglycerides and fatty acids. Mar Biol 117: 113–117.

[21] Yamashiro H, Oku H, Higa H, Chenen I, Sakai K (1999) Composition of lipid, was Esters, triglycerides and fatty acids and sterol in Okinawan coral. Comp Biochem Physiol 112B: 397–407.

[22] Davies PS (1991) Effect of daylight variations on the energy budgets of shallow-water corals. Mar Biol 108: 137–144.

[23] Pearse V, Muscatine L (1971) Role of symbiotic algae (zooxanthellae) in coral calcification, Biol Bull. 141: 350–363.

[24] Edmunds PJ, Davies PS (1986) An energy budget for Porites porites (Scleractinia). Mar Biol 92: 339–347.

[25] Spercer DP (1991) Effect of daylight variations on the energy budgets of shallow-water corals. Mar Biol 108: 137–144.

[26] Imbs AB, Demina OA, Demidkova DA (2006) Lipid class and fatty acid composition of boreal soft coral *Gersemia rubiformis* . Lipids 41: 721–725. 17069356 10.1007/s11745-006-5023-8

[27] Babcock RC, Heyward AJ (1986) Larval development of certain gamete-spawning scleractinian coral. Croal Reefs 5: 111–116.

[28] Yamashiro H, Oku H, Onaga K (2005) Effect of bleaching on lipid content and composition of Okinawan corals. Fish Sci 71: 448–453.

[29] Oku H, Yamashiro H, Onaga K, Iwasaki H, Sakai K (2002) Lipid distribution in branching coral Montipora digitata. Fish Sci 68: 517–522.

[30] Gorttoli AG, Rodrigues LJ, Juarez C (2004) Lipids and stable carbon isotopes in two species of Hawaiian corals, *Porites compressa* and *Montipora verrucosa*, following a bleaching event. Mar Biol 145: 621–631.

[31] Crossland CJ, Barnes DJ, Borowitzka MA (1980) Diurnal lipid and mucus production in the staghorn coral *Acropora acuminata* . Mar Biol 60: 81–90.

[32] Arai T, Kato M, Heyward A, Ikeda Y, Maruyama T (1993) Lipid composition of positively buoyant eggs of reef building corals. Coral Reefs 12: 71–75.

[33] Patton JS, Abraham S, Benson AA (1977) Lipogenesis in the intact coral *Pocillopora capitata* and its isolated zooxanthellae: evidence for a light-driven carbon cycle between symbiont and host. Mar Biol 44: 235–247.

[34] Oku H, Yamashiro H, Onaga K (2003b) Lipid biosynthesis from C-14 glucose in the coral Montipora digitata. Fish Sci 69: 625–631.

[35] Alava VR, Kanazawa A, Teshima S, Koshio S (1993) Effect of dietary phospholipids and n-3 highly unsaturated fatty acid on varian development of Kuruma prawn. *Nippon Suisan Gakkaishi* 59: 345–351.

[36] Ravid T, Tietz A, Khayat M, Boehm E, Michelis R, et al (1999) Lipid accumulation in the ovaries of a marine shrimp Penaeus semisulcatus (De Haan). J Exp Biol 202: 1819–1829. 10359684 10.1242/jeb.202.13.1819

[37] Moran AL, Manahan DT (2003) Energy metabolism during larval development of green and white abalone, *Haliotis fulgens* and *H. sorensem* . Biol Bull (Woods Hole) 204: 270–277. 10.2307/1543598 12807704

[38] Moran AL, Manahan DT (2004) Physiological recovery from prolonged ‘starvation’ in larvae of the Pacific oyster Crassostrea gigas. J Exp Mar Biol Ecol 306: 17–36.

[39] Sewell MA (2005) Utilization of lipids during early development of the sea urchin *Evechinus chloroticus* . Mar Ecol Prog Ser 304: 133–142.

[40] Zweytick D, Athenstaedt K, Daum G (2000) Intracellular lipid particles of eukaryotic cells. Biochim Biophys Acta 1469: 101–120. 10998572 10.1016/s0005-2736(00)00294-7

[41] Schafer WG, Schmidt H (1980) The anthozoan egg: Differentiation of internal oocytes structure. In: Developmental, Cellular Biology of Coelenterates. Tardent P, Tardent R, editors. Elsevier/North-Holland Biomedical Press. New York. 47–52.

[42] Harii S, Nadaoka K, Yamamoto M, Iwao K (2007) Temporal changes in settlement, lipid content and lipid composition of larvae of the spawning hermatypic coral *Acropora tenuis* . Mar Ecol Progr Ser 346: 89–96.

[43] Rodrigues LJ, Grottoli AG (2007) Energy reserves and metabolism as indicators of coral recovery from bleaching. Limnol Oceanogr 52: 1874–1882.

[44] Imbs A, Demidkova D, Latypov Y, Pham L (2007) Application of fatty acids for chemotaxonomy of reefbuilding corals. Lipids 42: 1035–1046. 17710463 10.1007/s11745-007-3109-6

[45] Treignier C, Grover R, Ferrier-Pagès C, Tolosa I (2008) Effect of light and feeding on the fatty acid and sterol composition of zooxanthellae and host tissue isolated from the scleractinian coral *Turbinaria reniformis*. Limnol. Oceanogr. 53: 2702–2710.

[46] Ulrich K (1994) Comparative animal biochemistry. Springer-Verlag.

[47] Latyshev NA, Naumenko NV, Svetashev VI, Latypow YY (1991) Fatty acids of reef-building corals. Mar Ecol Prog Ser 76: 295–301.

[48] Bell MV, Dick JR, Anderson TR, Pond DW (2007) Application of liposome and stable isotope tracer techniques to study polyunsaturated fatty acid biosynthesis in marine zooplankton. J Plankton Res 29: 417–422.

[49] Heyward AJ, Collins JD (1985) Growth and sexual reproduction in the scleractinian coral *Montipora digitata* (Dana). Aust J Mar Freshw Res 36: 441–446.

[50] Meyers PA (1979) Polyunsaturated fatty acids in coral: indicators of nutritional sources. Mar Biol Lett 1: 69–75.

[51] Holmer LE (1989) Middle Ordovician phosphatic inarticulate brachiopods from Vastergotland and Dalarna, Sweden. Fossils and Strata 26: 1–172.

[52] Mourente G, Odriozola JM (1990) Effect of brood stock diets on lipid classes and their fatty acid composition in eggs of gilthead sea bream (*Sparus aurata* L.). Fish Physiol Biochem 8: 93–101. 24221942 10.1007/BF00004436

[53] Jobling M, Jjohnsen HK, Pettersen GW, Henderson RJ (1995) Effect of temperature on reproductive development in arctic charr, *Salvelinus Alpinus* (L.). J therm Biol 20: 157–165.

